# Phosphorus uptake by *Zea mays* L. is quantitatively predicted by infinite sink extraction of soil P

**DOI:** 10.1007/s11104-014-2271-x

**Published:** 2014-09-24

**Authors:** Jakob Santner, Martina Mannel, Leigh D. Burrell, Christoph Hoefer, Andreas Kreuzeder, Walter W. Wenzel

**Affiliations:** Vienna, Department of Forest and Soil Sciences, Institute of Soil Research, University of Natural Resources and Life Sciences, Konrad-Lorenz-Strasse 24, 3430 Tulln, Austria

**Keywords:** Infinite sink extraction, Diffusive gradients in thin films, Phosphorus, Plant uptake

## Abstract

**Background and aims:**

Sink extraction of phosphorus from soils has been utilised to study soil P desorption kinetics and as index of plant availability, but not for quantitative prediction of P uptake by plants. Here we investigate the potential of a modified sink extraction method for determining P desorption kinetics and for quantifying plant available soil P.

**Methods:**

Modified diffusive gradients in thin films samplers were immersed in shaken soil suspensions for long-term extraction of soil P. Results were evaluated in terms of P desorption kinetics and compared to the P uptake of *Zea mays* L. and standard soil extracts.

**Results:**

In contrast to literature reports, four of the six studied soils only showed a rapid, but not a slowly desorbing P fraction. The quantity of P desorbed by long-term sink extraction not only showed the highest correlation to plant P uptake, but also matched plant P uptake quantitatively.

**Conclusions:**

Our data indicates that soils with only a fast desorbing P fraction might exist. Sink extraction methods have the potential to quantitatively predict plant P uptake. Furthermore, they could become valuable research tools for understanding P acquisition and might serve as a benchmark for calibrating soil P tests.

## Introduction

Infinite-sink methods have been proposed as alternatives to chemical extraction procedures for measuring reversibly adsorbed P in soils. In these methods, a phosphate-binding material is introduced, either into a soil suspension or into a soil paste of high water content. The resin binds phosphate and keeps the extractant (or porewater) phosphate concentration very low, shifting the equilibrium between surface-sorbed and dissolved P towards the dissolved phase. Through this process, phosphate is continuously desorbed from soil and captured on the binding material. This operating principle is very different to standardised batch soil extractions, where a soil sample is shaken with an extractant for a defined period of time. Batch extraction methods aim at establishing a quasi-equilibrium between P sorbed on the soil surfaces and P dissolved in the extractant. A true equilibrium is rarely reached, as this would usually require long extraction periods and large extractant volumes.

Amer et al. (1955) introduced the idea of infinite-sink extractions and reported a procedure that mixes anion resin beads with soil and water to extract P. However, separating soil and resin beads after extraction was tedious, therefore later studies used resin beads in membrane bags (Sibbesen 1977) and anion exchange membranes (Schoenau and Huang 1991) for resin P extraction. Instead of an ion exchange resin, van der Zee et al. (1987) used Fe-oxide coated paper strips, which also allowed for easy separation of sink and soil after extraction. Iron-oxides have the advantage of specifically binding phosphate with low competition from other anion species. However, a shortcoming of the Fe-oxide paper strips is that soil particles attach to the paper during the extraction (Chardon et al. 1996). These particles add P that is not desorbed from soil to the amount of P measured by the paper strips, which can account for up to 40 % of the total extracted P (Uusitalo and Yli-Halla 1999). To avoid this problem, Freese et al. (1995) filled dialysis membrane tubes with a suspension of hydrous ferric oxide and used these bags as infinite sinks (termed ‘Dialysis membrane tube – hydrous ferric oxide; DMT-HFO). The small pore size of the dialysis membrane ensures that only dissolved ions can pass the membrane and sorb onto the Fe-oxide, while particles are retained on the outer side of the membrane. Infinite-sink samplers of a similar configuration are used in the ‘diffusive gradients in thin films’ technique (DGT; Zhang et al. 1998). These samplers consist of a layer of ferrihydrite-containing polyacrylamide hydrogel, overlain by a pure hydrogel layer and a protective membrane housed in a plastic sampler moulding. Phosphate diffuses through the membrane and the diffusion layer and binds to the Fe-oxide contained in the resin gel. Due to the well-defined geometry of the DGT sampler, Fick’s law of diffusion can be used to calculate the time-averaged phosphate flux into the sampler as well as the time-averaged phosphate concentration at the sampler-medium interface (see material and methods section and Davison et al. 2007; Warnken et al. 2007). In DGT deployments on soil, the samplers are gently pushed into soil pastes that have a water content of 80–100 % of the maximum water holding capacity (Menzies et al. 2005). Like in DMT-HFO, the iron oxide is not in direct contact with the soil, but the phosphate ions diffuse to the sampler’s binding sites, thereby avoiding particle contamination.

In soils, these methods have mainly been used for: (1) the estimation of plant available P, and (2) the determination of P desorption kinetics from soil. The mechanistic similarity in the acquisition of P from soil solution by plant roots with P sorption by infinite sinks suggests that these methods should be highly suitable for soil testing. Both processes are controlled by diffusion of P towards the site of uptake and subsequent binding. In a conclusive review, Degryse et al. (2009) showed that DGT performs as well as, or better than, batch extractions as a nutrient test, if diffusion, and not mass flow or active P solubilisation such as the exudation of H^+^ or carboxylate anions, is controlling nutrient supply.

Several studies investigated the usefulness of infinite sink methods as indices for plant-availabe soil P. Resin P was found to significantly correlate with P uptake (Menon et al. 1989; Schoenau and Huang 1991; Tran et al. 1992), dry matter yield (Menon et al. 1989), and relative yield (Tran et al. 1992) on several occasions, while other studies found no correlation between resin P and plant characteristics (Mason et al. 2010, 2013). In cases where resin P showed a significant correlation, it was often not superior to other soil test methods (Mason et al. 2010; Menon et al. 1989).

Similar results were obtained for iron oxide paper strips. In a study by Menon et al. (1989) iron oxide paper P (P_i_) predicted the P uptake and relative yield of maize (*Zea mays* L.) better than soil extracts and resin P on soils fertilised with triple superphosphate, while with rock phosphate as a fertilizer the Bray 1, Bray 2 and double acid extracts performed better. A study by Hosseinpur and Safari Sinegani (2009) found equal or slightly weaker correlations between P_i_ and the P uptake of alfalfa (*Medicago sativa* L.) compared to those of standard extracts. A review of the application of Fe-oxide papers concluded that P_i_ is not necessarily superior to standard soil extraction in predicting P availability to plants, but is applicable in soils of different chemistry, i.e. in acid, alkaline and calcareous soils (Menon et al. 1996).

Recently, the DGT method was evaluated for soil testing in several studies. DGT was a better predictor than extraction and resin techniques of relative yield of tomato (*Solanum lycopersicum* L.; Menzies et al. 2005), wheat (*Triticum spp.*; Mason et al. 2010) and maize (Six et al. 2013), but not of rice (*Oryza sativa* L.; Six et al. 2013). In another study, DGT predicted the wheat response to fertilizer application effectively, but not as well as some of the tested extraction techniques (McBeath et al. 2007). Tandy et al. (2011) reported that DGT was a better predictor of P uptake by barley (*Hordeum vulgare* L.) compared to Olsen P and the soil solution P concentration. Isotope dilution studies demonstrated conclusively that DGT samples the soil P fraction that is accessible to plants by comparing the specific activity of plant and DGT P (Mason et al. 2013; Six et al. 2012). The specific activity of a range of soil extracts differed significantly from unity, indicating that extracts only partly measure the soil P that is plant-accessible, while the specific activity ratios of plant P and DGT P were not significantly different for most of the investigated soils. Resin methods performed slightly inferior compared to DGT in these two studies.

Apart from soil testing, sink methods have been used to study P desorption kinetics from soil. The amount of P desorbed from nine soils for up to 66 h was fitted to a kinetic model.

1 $$ Q\mathrm{t}=Q \max\;\left(1-{e}^{-k\;t}\right) $$

where *Q*_t_ is the amount of P desorbed at time *t*, *Q*_max_ is the maximum desorbable amount of P and *k* is the desorption rate constant (van der Zee et al. 1987). In long-term extraction experiments (up to 1,600 h) on 44 soils, Lookman et al. (1995) found that the extraction data better fitted a model that combined a fast and a slowly desorbing P pool.

2 $$ {Q}_t={Q}_1\left(1-{e}^{-{k}_1t}\right)+{Q}_2\left(1-{e}^{-{k}_2t}\right) $$

where the indices 1 and 2 denote the two pools, respectively. P desorption from the 44 soils studied generally had a fast and slow desorption phase that continued to desorb for at least 1,600 h. Several other studies found similar P desorption behaviour in soils (de Jager and Claassens 2005; Maguire et al. 2001; Taddesse et al. 2008c).

Sink methods have been used for a long time for P analysis in soils. However, the reversibly adsorbed P quantity that sink methods measure has not been related to plant growth characteristics to date. This work sets out to employ DGT samplers as long-term infinite sinks for P, as these are commercially available and their use by researchers is constantly increasing. The amounts of P extracted from six experimental soils during a 30-day period are compared to plant P uptake and the P extracted by standard soil P tests.

## Material and methods

### Soils

All experiments were conducted using six soils that differ in pH, texture, carbonate and total P content and P solubility (Table 1, 2). The soils originated from four locations in Austria (Forchtenstein, Horn, Aigen and Trautenfels), one location in Germany (Blankenstein) and one location in Spain (Santomera). The Forchtenstein soil is a forest soil which never received fertiliser. The Santomera soil originates from a research station and did not receive fertiliser since 2001. The other four soils are arable soils used in crop rotation schemes. They received liquid manure (Aigen, Blankenstein, Trautenfels), mineral P fertilisers (Aigen, Trautenfels), compost (Trautenfels) and manure (Aigen) during 2003–2011. The Horn soil was also amply fertilised with P before excavation, however no details on the P fertilisation history of this soil are available. All experimental soils were air dried and sieved to <2 mm before use.

**Table 1:** Soil properties

Soil	pH CaCl_2_	sand	silt	clay	soil textural class ^a^	carbonate content ^b^	C_org_	P_AR_^c^	P_org_	P_AAO_^d^	P saturation (α)	Fe_AR_	Fe_CBD_^e^	Fe_AAO_	Al_AR_	Al_CBD_	Al_AAO_
		g kg^−1^	g kg^−1^	g kg^−1^		g kg^−1^	g kg^−1^	mg kg^−1^	mg kg^−1^ (% P_AR_)	mg kg^−1^		g kg^−1^	g kg^−1^	g kg^−1^	g kg^−1^	g kg^−1^	g kg^−1^
Forchtenstein	4.0	443	374	182	L		25.4 (1.1)	412 (7.9)	225 (54.6)	233 (32.1)	0.048 (0.007)	43.4 (7.4)	6.44 (0.87)	5.03 (0.79)	54.1 (0.5)	1.41 (0.33)	1.80 (0.21)
Horn	5.7	422	336	243	L		11.8 (0.4)	2,300 (36.2)	323 (14.1)	478 (96.7)	0.323 (0.020)	48.6 (5.4)	5.40 (0.91)	1.44 (0.22)	86.9 (2.6)	1.14 (0.14)	0.59 (0.08)
Blankenstein	6.2	51	745	203	SiL		19.1 (0.8)	782 (29.5)	329 (42.0)	342 (67.1)	0.138 (0.006)	30.0 (2.5)	12.4 (1.77)	2.90 (0.57)	39.3 (2.2)	1.82 (0.19)	0.76 (0.12)
Aigen	7.1	498	329	173	L	16.9	13.9 (0.4)	1,600 (91.2)	200 (12.5)	441 (80.0)	0.176 (0.017)	29.9 (5.9)	11.8 (2.15)	3.45 (0.35)	65.4 (1.8)	1.07 (0.25)	0.51 (0.04)
Trautenfels	7.3	285	572	143	SiL	53.1	23.5 (1.5)	870 (14.5)	231 (26.5)	295 (14.9)	0.220 (0.010)	22.2 (0.2)	12.9 (0.84)	1.68 (0.10)	20.0 (0.3)	1.06 (0.07)	0.35 (0.02)
Santomera	7.8	219	475	306	CL	499	7.3 (0.4)	378 (10.1)	74.6 (19.7)	131 (18.2)	0.234 (0.012)	24.1	10.6 (4.57)	0.28 (0.03)	26.2	0.72 (0.07)	0.35 (0.04)

^a^ L loam, SiL silt loam, CL clay loam according to FAO (2006). ^b^ as CaCO_3_ equivalent. ^c^ *aqua regia* extractable. ^d^ acid ammonium oxalate extractable. ^e^ citrate bicarbonate dithionite extractable. Values in parentheses are the standard deviation of the mean (*n* = 3) unless otherwise stated.

**Table 2:** Extractable soil P fractions

Soil	CAL	Colwell P	Ca (NO_3_) _2_	*c*_DGT_
	mg kg^−1^	mg kg^−1^	μg kg^−1^	μg L^−1^
Forchtenstein	1.6 (0.1)	15.8 (0.8)	202 (5)	23.5 (1.2)
Horn	99.7 (3.8)	88.3 (1.1)	1,420 (14)	175 (1.8)
Blankenstein	46.0 (0.2)	42.3 (0.6)	564 (11)	111 (3.3)
Aigen	93.9 (2.0)	49.5 (0.8)	1,330 (41)	238 (9.8)
Trautenfels	164 (5.3)	94.4 (3.8)	1,460 (22)	260 (3.0)
Santomera	38.1 (0.7)	12.1 (0.1)	109 (9)	11.9 (0.5)

Values in parentheses are the standard deviation of the mean (*n* = 4 for *c*_DGT_, *n* = 3 for batch extractions).

### Soil P analyses

Three soil P extractions procedures, calcium acetate – lactate P (CAL; OENORM L1087 1993; Schüller 1969), Colwell P (Colwell 1963) and Ca (NO_3_) _2_ extraction (Muhammad et al. 2012), were performed.

CAL is the Austrian norm for soil P testing. Five grams of soil were shaken in 100 mL of a solution containing 62 mmol L^−1^ calcium lactate, 56 mmol L^−1^ calcium acetate and 391 mmol L^−1^ acetic acid, with the pH of the solution adjusted to pH 4.1, for 2 h. Colwell P is a widely used P test and is the Australian standard for agricultural P testing. For this procedure, 1 g of soil was shaken with 50 mL of 0.5 mol L^−1^ NaHCO_3_ solutions adjusted to pH 8.5 using 1 mol L^−1^ NaOH for 16 h. Dilute salt extracts are commonly used to extract the labile soil P at an electrolyte concentration similar to that of soil pore water. In this study, we extracted 5 g of soil in 25 mL of 5 mmol L^−1^ Ca (NO_3_) _2_ solutions for 2 h.

All extractions were undertaken with an overhead shaker at 20 rpm. The extracts were filtered with paper filters (grade 14/N; Munktell & Filtrak GmbH, Bärenstein, GER). In addition, the Ca (NO_3_) _2_ extracts were centrifuged at 2,500 × g for 3 min before filtration. The Colwell extracts were acidified before analysis by adding a drop of p-nitrophenol to the extract and titrating the sample with 2.5 mol L^−1^ H_2_SO_4_ until the sample colour changed from yellow to colourless.

Total organic soil P (P_org_) was determined as the difference between 0.5 mol L^−1^ H_2_SO_4_ extractable P in combusted and uncombusted soil samples (Kuo 1996).

Amorphous Fe- and Al-(oxy) hydroxides were determined in acid ammonium oxalate at pH 3 (Loeppert and Inskeep 1996). The degree of P saturation of the amorphous Fe- and Al-(oxy) hydroxides, α, is defined as [P_AAO_]/[Fe_AAO_ + Al_AAO_] (van der Zee et al. 1987), with P_AAO_ being the amount of P released from these minerals, as determined in the same extract. Where required, calcium carbonate was removed prior to this extraction by washing the soil in ammonium acetate at pH 5.5.

Total free Fe and dithionite-extractable Al, which is usually considered the Al contained in crystalline Al-(oxy) hydroxides (Bertsch and Bloom 1996), were determined using the citrate-bicaronate-dithionite (CBD) method (Loeppert and Inskeep 1996).

For the determination of *aqua regia*-soluble (pseudo-) total soil P, Fe and Al, 0.5 g of soil were digested in 6 mL *aqua regia* (10.2 mol L^−1^ HCl : 15.5 mol L^−1^ HNO_3_; 3:1; v:v) using a Multiwave 3,000 (Anton Paar, Hertford Herts, UK) microwave digestion system. The digests were filtered using paper filters.

### DGT samplers

Diffusive (0.8 mm thick) and ferrihydrite resin gels (0.4 mm thick) were produced according to published procedures (Santner et al. 2010; Zhang and Davison 1999). DGT samplers were obtained from DGT Research Ltd (Lancaster, UK). After DGT application, the samplers were retrieved and rinsed with lab water (18 MΩ cm, prepared by a Millipore Elix 3 water purification system). The samplers were then carefully opened, the ferrihydrite gel disk retrieved and eluted in 10 mL of 0.25 mol L^−1^ H_2_SO_4_. If low P recovery was expected, gels were eluted in 2 mL of 0.25 mol L^−1^ H_2_SO_4_. Ferrihydrite dissolves in 0.25 mol L^−1^ H_2_SO_4_, leading to a recovery of 100 % of the sorbed P (Santner et al. 2010).

### Conventional DGT deployments on soil

For conventional DGT sampling on soil, DGT units were filled with a 2.5 cm diameter ferrihydrite gel disk followed by a disk of polycarbonate membrane (pore size 0.2 μm, thickness 10 μm; Nuclepore, GE Healthcare, Freiburg, GER), a disk of diffusive gel and a layer of protective membrane (pore size 0.45 μm, thickness 140 μm; Supor, Pall GmbH, Dreieich, GER). Soil was mixed with water to obtain a paste at a water content of 90 % of the maximum water holding capacity of the soil. The soil pastes were incubated for 24 h at 20 °C to allow for the equilibration of the dissolved and sorbed fractions of soil phosphorus prior to DGT application. After this period, the paste was carefully smeared onto the DGT samplers. The samplers were incubated for 24 h at 20 °C for P uptake. Results are expressed as *c*_DGT_ values according to

3 $$ {c}_{\mathrm{DGT}}=\frac{M\varDelta g}{DAt} $$

where *c*_DGT_ is the time-averaged soil-solution P concentration at the sampler-soil interface, *M* is the mass of P accumulated by the sampler, *Δg* is the diffusion layer thickness, *D* is the diffusion coefficient in the diffusion layer, *A* is the sampling area and *t* is the deployment time (Davison et al. 2007; Warnken et al. 2007).

### Quantification of labile P using DGT samplers as infinite sinks

**Fig. 1:**
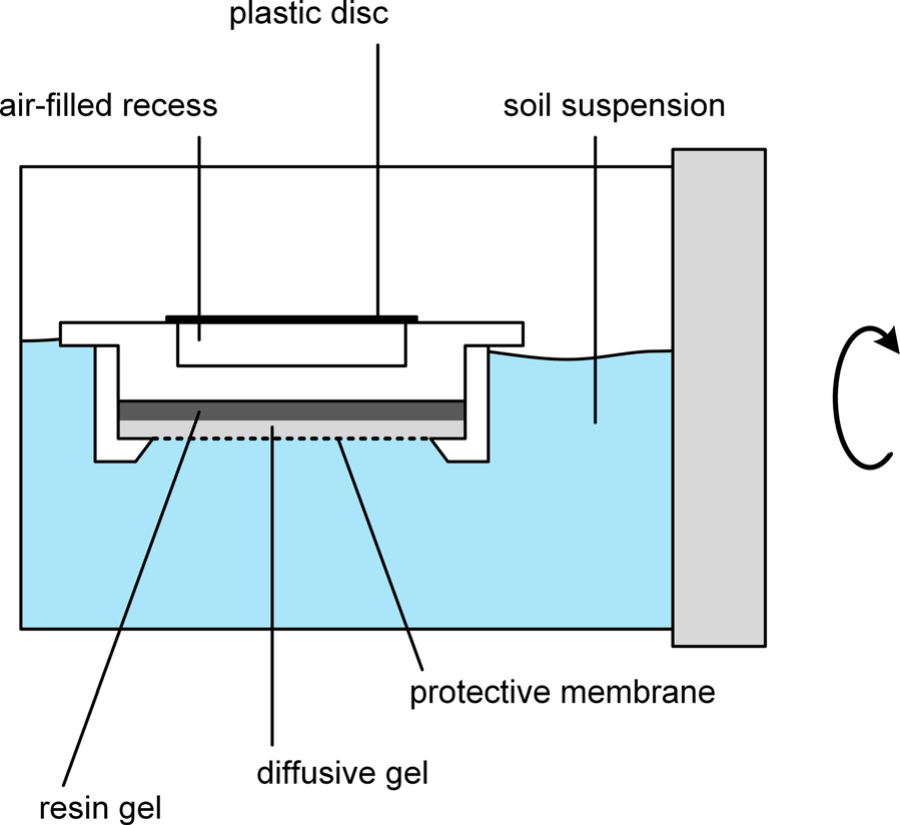
Schematic of the long-term P extraction setup. A DGT sampler was kept afloat in the extractant-soil suspension by gluing a plastic disc across an existing recess in the standard DGT sampler geometry. The bottle containing the suspension and the sampler was gently shaken in a ‘rolling’ mode at 2–3 rpm, as indicated by the arrow. In this way, the sampler ‘swims’ on the extractant with the P-sorbing side of the sampler being continuously exposed to the suspension. Conventional DGT samplers would sink in the suspension and would be continuously overturned during the extraction. Schematic not to scale

For the quantitative extraction of the inorganic, labile P pool from the experimental soils DGT samplers were exposed to soil suspensions for up to 30 days. Standard DGT samplers sink in water, therefore we entrapped an air bubble in the samplers by gluing a plastic disc across a recess in the DGT geometry (Fig. 1). This air bubble ensured that the DGT device drifted on the extraction solution during the depletion experiment with the sampling window facing into the extraction solution. To maximise the P flux into the sampler, we used a 0.01 mm thick polycarbonate membrane as a diffusive layer. Underneath this membrane the ferrihydrite gel and a 0.8 mm thick plastic spacer disk were placed. This gel layer setup was chosen to achieve complete extraction of labile soil P as quickly as possible, as a thin diffusion layer increases the P concentration gradient, and thereby the P flux from the outer medium into the resin gel.

Studying P desorption kinetics in long-term desorption experiments requires the elimination of microbial or redox-induced mobilisation of P. Freese et al. (1995) used chloroform as a biocidal treatment, however, it is not clear if other studies (de Jager and Claassens 2005; Maguire et al. 2001; Taddesse et al. 2008b; van der Zee et al. 1987) did so as well. In this study we use NaN_3_ as biocide.

For the quantitative extraction of labile P from soil, extraction solutions containing 10 mmol L^−1^ Ca (NO_3_) _2_ as background electrolyte, 30 mmol L^−1^ NaN_3_ as biocide and 30 mmol L^−1^ pH buffer, were prepared. The pH of the extraction solutions was buffered for each soil individually using suitable pH buffering agents at the soil’s pH measured in 10 mmol L^−1^ CaCl_2_ suspensions. Benzoic acid was used for the Forchtenstein soil, 2-(*N*-morpholino) ethanesulfonic acid (MES) for the Horn, Blankenstein and Aigen soils and 3-(*N*-morpholino) propanesulfonic (MOPS) acid for the Trautenfels and Santomera soils. pH adjustment was conducted using either dilute NaOH or dilute HCl. Sodium azide inhibits O_2_ respiration by irreversibly binding to cytochrome-c-oxidase. Its addition to the extraction solution ensured O_2_ concentrations of about 6 mg L^−1^ (measured using an aquarium O_2_ testing kit) throughout the 30-day extraction period. Anaerobia in the soil suspensions was thereby prevented, minimizing possible artefacts caused by the reductive dissolution of Fe-(oxy) hydroxides and the concomitant release of adsorbed P. Fifty mL extractant was transferred into acid-washed 120 mL bottles, along with 0.5 g of soil. This wide extractant/soil ratio was chosen to keep the P concentration in the extractant low and to favour P desorption from soil surfaces. One modified DGT sampler was placed into the extraction solution. The bottles were closed and shaken in an overhead shaker in a ‘rolling’ mode at 2-3 rpm for 1, 2, 3, 4, 7, 10, 14, 18, 22, 26 and 30 days. Results were fitted to Eqs. 1 and 2.

### Plant experiment

Subsamples of all soils were amply fertilised with N, K, Mg, S and Zn (in mg kg^−1^; NH_4_NO_3_ 181, KCl 145, MgCl_2_ × 6 H_2_O 134, ZnSO_4_ × 7 H_2_O 34) to ensure that plant growth was not limited by these nutrients. Fertiliser addition was carried out by adding the nutrient salts as solutions to individual portions of soil, allowing the portions to dry, gently crushing the dried soils to powders and mixing them back into the respective subsample.

*Zea mays* cv. ‘Die Saskia’ (DKC 3476) seeds were germinated in petri dishes on wet tissue paper for 3 days. Pots were filled with 1 kg of fertilised soil. Two maize seedlings were planted in each pot. Pots were watered with 300 mL water per pot at the beginning of the experiment. The soil surface was covered with a 1-cm layer of polyethylene granulate to reduce evaporation. Five days after planting the smaller seedling was gently pulled out of the soil. The maize plants were grown in a greenhouse for 7 weeks, during which period the pots were randomised weekly. Humidity in the greenhouse cabin was maintained at 60 %, the day/night cycle was set to 16/8 h. Below a solar photon flux density of 370 μmol m^−2^ s^−1^ additional artificial lighting of ~ 500 μmol m^−2^ s^−1^ was provided. The plants were watered with approximately 50 mL tap water per day.

At harvest, shoots were cut and washed with lab water. Roots were gently separated from the soil and rinsed with lab water. The roots were then washed for 5 min in a 10 mmol L^−1^ CaCl_2_ solution using an ultrasonic bath to remove remaining soil particles. Shoots and roots were then dried at 65 °C for 3 days and ground to a fine powder. Subsequently, 0.2 g of ground plant material were digested in a mixture of 5 mL HNO_3_ (15.5 mol L^−1^) and 1 mL H_2_O_2_ (30 %) in a microwave digestion system.

### Analyses

Soil extracts, plant and soil digests, and ferrihydrite gel eluates, were analysed for P using a molybdate blue procedure (Zhang et al. 1998) after appropriate dilution on a Hitachi U-2000 UV/VIS spectrophotometer (Hitachi High-Technologies Corporation, Tokyo, JP). Fe, Al and P in the ammonium-oxalate extracts, as well as Fe and Al in the citrate-bicarbonte-dithionite extracts and the soil digests, were measured by ICP-OES (Optima 8300, Perkin Elmer, Waltham, MA, USA). Certified reference materials (digestion), in-house reference materials (extractions) and digestion/method-blanks were used for quality control of the analyses.

## Results

### Soil P desorption kinetics

**Fig. 2:**
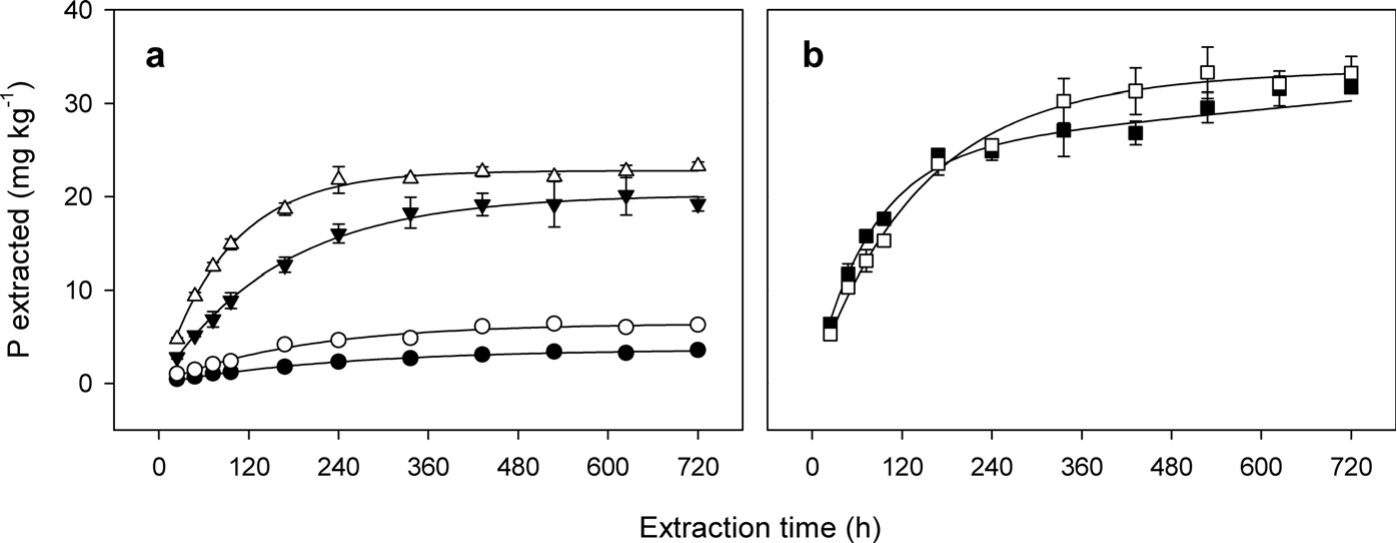
Extracted soil P during the 30-day extracting period. Symbols denote the extracted amounts of P while lines are the fits of Eqs. 1 and 2 to the experimental data. The left pane shows the desorption data that followed only one desorption kinetic (Eq. 1), while the desorption data in the right pane have two P fractions that desorb with different rate constants (Eq. 2). The symbols and lines correspond to the six experimental soils in the following order (*bottom to top*). **a** Santomera, Forchtenstein, Blankenstein, Aigen. **b** Trautenfels, Horn. Error bars are the standard deviation of the mean (*n* = 3)

The long-term P extraction experiment yielded saturation-type P desorption curves (Fig. 2). The maximum desorbed amounts of P (*P*_max_) ranged from 3.57 to 33.3 mg kg^−1^ (Table 3) and were obtained after extraction periods of 528–720 h. Desorption curves visually suggest that a plateau in the P extractability is reached within the 720-hour desorption period for all soils except Trautenfels. Fits of Eq. 1 explained the desorption data of four of the soils (Forchtenstein, Santomera, Blankenstein, Aigen) well. The model containing two desorbing P fractions (Eq. 2) did not fit these datasets within valid parameter ranges (*Q*_2_ and *k*_2_ estimates were implausibly small and large, respectively). By comparing *P*_max_ to the fitted amount of reversibly adsorbed P (*Q*_1_), it seems that in these four soils, P desorption is largely finished after 720 h. In the remaining two soils, Trautenfels and Horn, it is apparent that two P fractions contribute to the total P desorption from soil.

**Table 3:** Parameters of the kinetic desorption models (Eqs. 1 and 2) obtained by fitting the data of the long-term extraction experiment

Soil	*P*_max_	*Q*_1_	*k*_1_	*Q*_2_	*k*_2_
	mg kg^−1^	mg kg^−1^	h^−1^	mg kg^−1^	h^−1^
Forchtenstein	6.41 (0.41)	6.46	0.00537		
Horn	33.3 (2.73)	32.2	0.00716	105	0.000016
Blankenstein	20.2 (2.12)	20.2	0.00619		
Aigen	23.3 (0.43)	22.8	0.01085		
Trautenfels	31.8 (0.68)	24.6	0.01298	83.7	0.000098
Santomera	3.57 (0.29)	3.70	0.00415		

Values in parentheses are the standard deviation (*n* = 3) of the mean.

### The correlation of soil P extracts, soil P saturation and soil Al and Fe fractions to *P*_max_

Table 2 gives an overview on the P extracted from soil by standard chemical extracts and the DGT method. Colwell and CAL P are in a similar range (1–160 mg kg^−1^), while Ca (NO_3_) _2_ is much lower (100–1,500 μg kg^−1^). The range of *P*_max_ values measured for our soils is lower than Colwell and CAL P, but higher than Ca (NO_3_) _2_. As the *c*_DGT_ values cannot be related to an amount of soil from which P is released, they are presented as time-averaged concentrations at the soil-sampler interface. All extracted P concentrations are significantly linearly correlated to *P*_max_ (Table 4).

**Table 4:** Product–moment correlation coefficients of soil P fractions with extracted P (*P*_max_) and plant P uptake (*n* = 6)

	*P*_max_	plant P uptake
*P*_max_		0.994 **
*c*_DGT_	0.894 *	0.849 *
CAL P	0.855 *	0.834 *
Colwell P	0.973 **	0.973 **
Ca (NO_3_) _2_ P	0.955 **	0.936 **

* indicates significant correlation at the *P* ≤ 0.05 and ** at the *P* ≤ 0.01 level.

The P saturation, α, varies in a range between 0.05 and 0.32 for the experimental soils (Table 1). A significant correlation of the P saturation to *P*_max_ was found (R = 0.94, *P* ≤ 0.05) if the Santomera soil was excluded from the analysis. Exluding this soil was necessary and justified because the α value for the Santomera soil (α = 0.24) is astonishingly high, considering that all other P analyses reveal that Santomera is a very low P soil. The reason for this apparent inconsistency might be due to the analytical procedure and the high CaCO_3_ content of Santomera. Before determining Fe_AAO_, Al_AAO_ and P_AAO_, the carbonate is dissolved in a pH buffer at pH 5.5 and is subsequently washed off. The P that is liberated from Ca-P in this process might readily adsorb to Fe- and Al-(oxy) hydroxides during the washing step and subsequently be measured as P_AAO_. For this reason, Santomera was excluded when the P saturation was taken into account in data interpretation.

Potential correlations of *P*_max_ and plant P uptake to soil Al and Fe fractions (acid ammonium oxalate-, citrate-bicarbonat-dithionite-, and *aqua regia*-soluble) were investigated, however no significant correlations were found.

### Plant growth

**Fig. 3:**
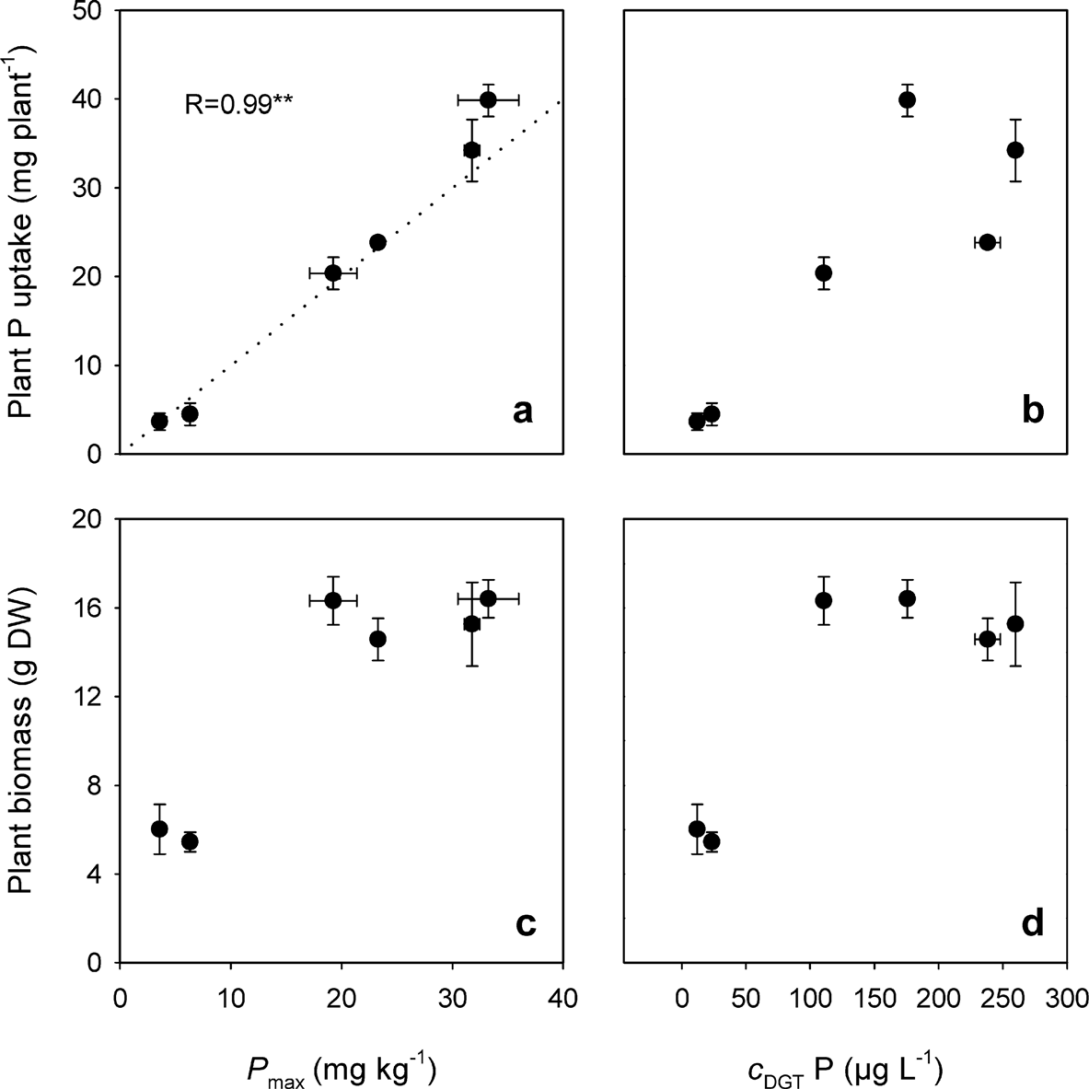
Correlation of DGT-measured P (*P*_max_, *c*_DGT_) with plant biomass and plant P uptake. For plant P uptake, total plant P was corrected for seed P content. R is the product–moment correlation coefficient, ** indicates a significant correlation at the *P* ≤ 0.01 level. The dotted line is the 1–1 line. Error bars represent the standard deviation of the mean (*n* = 4 for plant parameters and *c*_DGT_, *n* = 3 for *P*_max_)

The biomass yield of the maize plants shows two groups, one group with relatively little biomass production on the Forchtenstein and Santomera soils (average plant biomass of 6.0 and 5.4 g DW plant^−1^), and a second with 14.6–16.4 g DW plant^−1^ for the remaining four soils (Table 5, Fig. 3). The phosphorus concentrations in the maize shoots are below 900 mg kg^−1^ in the plants of the first group and between 1,320 and 2,610 mg kg^−1^ in the plants of the second group.

**Table 5:** Shoot and root characteristics of *Zea mays* L. obtained in the plant experiment

Soil	root biomass (DW)	shoot biomass (DW)	root P concentration	shoot P concentration	plant P uptake ^a^
	g	g	mg kg^−1^	mg kg^−1^	mg plant^−1^
Forchtenstein	0.95 (0.33)	5.07 (0.82)	850 (79)	891 (67)	4.5 (1.3)
Horn	2.37 (0.31)	12.9 (1.6)	3,171 (553)	2,600 (332)	39.9 (1.8)
Blankenstein	2.60 (0.21)	13.7 (1.0)	1,200 (40)	1,320 (68)	20.4 (1.8)
Aigen	2.57 (0.32)	12.0 (0.7)	1,370 (138)	1,760 (160)	23.8 (0.0)
Trautenfels	3.24 (0.14)	13.2 (0.8)	1,970 (51)	2,170 (140)	34.2 (3.5)
Santomera	0.92 (0.10)	4.52 (0.37)	761 (84)	824 (121)	3.7 (1.0)

^a^ corrected for the P content of the maize seed (0.797 mg seed^−1^). Values in parentheses are the standard deviation of the mean (*n* = 4).

### The correlation of plant P uptake to sink-extracted P

The amounts of P extracted with conventional soil P tests (CAL, Colwell, Ca (NO_3_) _2_) and *c*_DGT_ all correlate closely with plant P uptake (Table 4), however the strongest correlation was obtained between *P*_max_ derived from the long-term desorption experiment and plant P uptake. Moreover, the absolute amounts of *P*_max_-P and plant P uptake are almost perfectly equal (Fig. 3a).

## Discussion

### Soil P desorption kinetics

The amounts of the fast desorbing reversibly adsorbed P fraction *Q*_1_ (3.7 – 32.2 mg kg^−1^; Table 3) determined in this study are in the lower range of reported values (Koopmans et al. 2001; Lookman et al. 1995; van der Zee et al. 1987). In contrast to the cited references, which used acidic, sandy and P-rich soils throughout, the soils used in this study are silt loams, clay loams and loams, which generally have a higher P binding capacity than coarser soils due to their higher specific surface area.

The desorption rate constants *k*_1_ measured in our study are in the low range of the values determined by Lookman et al. (1995), but higher than those of Koopmans et al. (2001) (Table 3, 6). Both studies used the DMT-HFO technique. Rate constants measured with Fe-oxide papers (van der Zee et al. 1987), however, are ~20–30 times higher than those of our study. The adherence of soil particles to Fe-oxide papers overestimates P desorption rate constants (Freese et al. 1995), which is not the case for the DMT-HFO technique and which should also not affect the rate constants measured by our DGT sink technique. Moreover, desorption rate constants measured in soil suspensions may be overestimated as the disaggregation of soil particles can lead to increased access to intra-particle sorption sites (Nye and Staunton 1994; Staunton and Nye 1989). The *k*_2_ values measured in our study and in that of Lookman et al. (1995) are lower than a theoretically derived *k*_2_ value (Ptashnyk et al. 2010), indicating that overestimations of the experimentally determined *k*_2_ are unlikely. The desorption rate constants estimated by Ptashnyk et al. (2010) consider desorption from the soil surface (*k*_1_) and desorption from the surface after intra-particle diffusion to the surface (*k*_2_), but not transport through the external solution. However, transport through the suspension is a component in the extraction setups discussed here, which is reflected in the measured values being lower than the theoretical ones (Table 6). In general, diffusion and not phosphate desorption from soil surfaces may be rate-limiting for the P resupply to plant roots or mycorrhizas in natural, structured soils (Nye and Staunton 1994).

**Table 6:** Value ranges for desorption rate constants determined in this and other studies

Estimation approach	Source	*k*_1_		*k*_2_	
		h^−1^		h^−1^	
		max	min	max	min
long-term DGT	this study	0.0130	0.0042	0.00010	0.00002
DMT-HFO	Lookman et al. (1995)	0.0624	0.0097	0.00068	0.00007
DMT-HFO	Koopmans et al. (2001)	0.0016	0.0015	nr	
Fe-oxide paper *	van der Zee et al. (1987)	0.353 *	0.079 *	nr	
modelling	Ptashnyk et al. (2010)	instantaneous		0.0011	

* potentially overestimated by particle contamination during sampling (Freese et al. 1995)

nr: not reported

Our observation of only one P releasing pool in four of the experimental soils is contrary to previous studies which generally reported two desorbing fractions (de Jager and Claassens 2005; Lookman et al. 1995; Maguire et al. 2001; Taddesse et al. 2008a). Lookman et al. (1997) showed that the fast desorbing P pool consists of amorphous, soluble Ca-phosphates and protonated, loosely adsorbed P that is associated to Al surfaces. It seems feasible that loosely adsorbed Fe-P also contributes to this fraction, but this was not measured in their NMR-based study. The slowly desorbing P pool most likely consists of P sorbed to intra-particle surfaces and poorly soluble phosphate minerals (Barrow 1986; McDowell and Sharpley 2003).

The Forchtenstein soil is a non-agricultural soil that never received P fertiliser and the Santomera soil was not fertilised since 2001. In contrast, Blankenstein and Aigen are fertilised, agricultural soils. Forchtenstein and Blankenstein are non-calcareous, Aigen has low carbonate content and Santomera is highly calcareous (Table 1). In all of these contrasting soils no slowly desorbing P fraction was detected. In Forchtenstein, a possible explanation for this lack of slow P desorption might be the low P saturation (Table 1) of the P sorption sites in this soil. If only a small fraction of the binding sites is P-saturated, the remaining sites might act as strong sinks that prevent the diffusion of P into soil particles, thereby keeping the slowly desorbing P fraction small. However, in the Blankenstein and Aigen soil the P saturation does not seem to be responsible for the existence of only one desorbing fraction, as their α is similar to that of the Trautenfels soil. In the highly calcareous Santomera, dissolved phosphate will quickly precipitate to Ca-phosphate, which might prevent intra-particle P diffusion and the formation of a detectable slowly desorbing P pool in a similar way.

A further complication is that only few of the cited papers clearly state if a biocide was added to the extractant for preventing microbial degradation of organic P and anoxia-induced reductive dissolution of P-bearing Fe-(oxy) hydroxides. If no biocide was present for the reported extended extraction periods (up to 1,600 h), microbially released P could be interpreted as slow-release reversibly adsorbed P. The biocidal treatment in our study succeeded in maintaining the extractant oxic for 30 days, so microbial P solubilisation and dissolution of P sorption sites can be ruled out for this study.

Taken together, the reason for only measuring dual-phase desorption kinetics in two out of six soils in this study is unclear. However, the fact that other researchers found a slowly and a fast desorbing fraction in all investigated soils using similar infinite sink extraction techniques indicates, that the lack of a slowly desorbing P fraction is not related to artefacts associated with the extraction procedure.

### Plant P uptake from soil

For the Forchtenstein and Santomera soils the low P concentrations and the decreased biomass production are clearly related to the low soil P levels (Table 1, 2). In these plants we also observed purple discoloration of the leaves, indicating P deficiency. The plateau in plant biomass production on the remaining four soils indicates that plant growth is not limited by P supply in these plants, even though the shoot P concentrations on the Blankenstein (1,320 mg kg^−1^) and the Aigen (1,760 mg kg^−1^) soils are low compared to the P requirement thresholds of ~ 2,000 mg kg^−1^ reported for agricultural crops (Bergmann 1993). On the Trautenfels and Horn soils, shoot P concentrations were higher than this critical level. No discolorations were present in any leaves of the plants from these four soils.

**Fig. 4:**
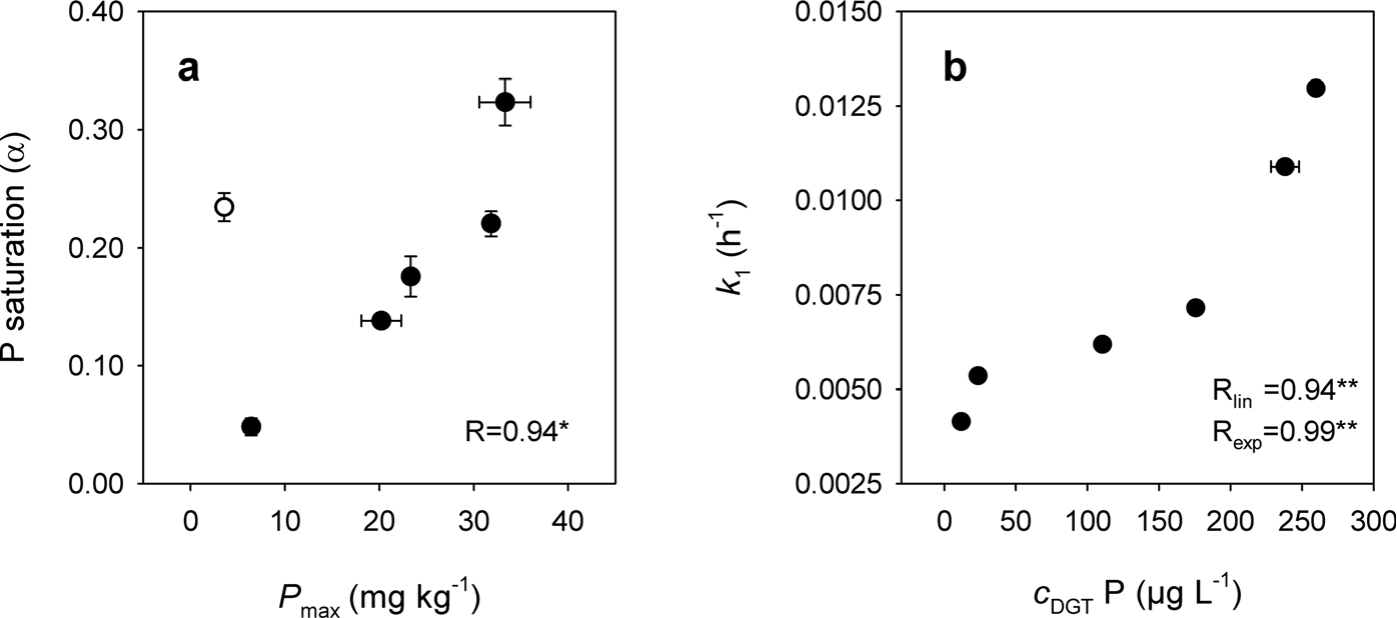
**a** Correlation of *P*_max_ and soil P saturation (α). Filled symbols are the non- and low-calcareous soils, the open point is the Santomera soil (499 g kg^−1^ CaCO_3_; see Table 1). The R value was calculated for the five filled points only as α determined in non/low calcareous soils and highly calcareous soils might not be comparable (See Results section for details). **b** Relationship of *c*_DGT_ values and desorption rate constants of the quickly desorbing P fraction (*k*_1_). The correlations of the variables using a linear model and a simple exponential model of the form *y* = *ae* ^*bx*^ + *c*, where *a*, *b* and *c* are fitting parameters were tested. R_lin_ and R_exp_ denote the respective product–moment correlation coefficient, * indicates a significant correlation at the *P* ≤ 0.05 level and ** indicates a significant correlation at the *P* ≤ 0.01 level. Error bars represent the standard deviation of the mean (*n* = 4 for *c*_DGT_, *n* = 3 for α and *P*_max_)

Figure 3a shows the relationship of *P*_max_ and plant P uptake. *P*_max_ data is given in mg kg^−1^ in this plot, while P uptake is given in mg plant^−1^. However, as the maize plants ‘extracted’ P from one kg of soil (1 kg soil per pot), these values also represent P extracted from soil in mg kg^−1^. It is important to note that the root systems of the plants were well developed compared to the limited amount of soil in the pots. The very good agreement in absolute numbers of *P*_max_ with plant P uptake therefore indicates that the plants mainly forage P from the reversibly adsorbed P fraction. The strong correlation of the P saturation, α, with *P*_max_ (Fig. 4a) and with plant P uptake (data not shown) further indicates that the degree of sorption site P loading is important in determining the quantity of desorbable P. This correlation shows that the P saturation of (oxy) hydroxides of Fe and Al controls the amount of desorbable P in noncalcareous and low-calcareous soils, which in turn limits the amount of P available to plants.

Plant P uptake and *P*_max_ deviate by more than 10 % only in two of the experimental soils. The plants extracted only ~ 70 % of *P*_max_ -P from low-P Forchtenstein soil, thus less P was available to the plants than to the abiotic infinite sink. Competition for soil P by microorganisms in the plant experiment could possibly explain this deviation, as the sink extraction was undertaken in sterile conditions. In Horn soil, the maize plants took up 21 % more P than predicted by *P*_max_.

While *P*_max_ and P uptake are significantly linearly correlated, the plot of *c*_DGT_ P vs. P uptake does not suggest a linear correlation (Fig. 3b), although the linear correlation is statistically significant. The datapoints for the Santomera, Forchtenstein, Blankenstein and Horn soils lie on a straight line in this plot, whereas the Aigen and Trautenfels points shifted towards higher *c*_DGT_ values. Here the different extraction periods for *P*_max_ and *c*_DGT_, and P desorption kinetics, have to be taken into account. The maize plants took up P for seven weeks. The long-term sink extractions showed that the period necessary for a strong sink to largely deplete the fast desorbing P fraction was about 3 weeks (Fig. 2), thus the P amounts taken up by the plants are determined by the extractable P quantity (*P*_max_). In contrast, the *c*_DGT_ values were measured in 24 h applications on fresh (undepleted) soil pastes. During this short DGT exposure soil P is not greatly depleted, hence the amount of P sampled is largely determined by the rate of P supply to the DGT samplers, i.e. the rate of P desorption from soil. A plot of *k*_1_ vs. *c*_DGT_ shows that these two parameters are correlated and fit a linear model (R = 0.94; R^2^ = 0.77, *P* ≤ 0.01; Fig. 4b). However, the plot rather suggests a non-linear correlation of *k*_1_ to *c*_DGT_ which also fits the data well (R^2^ = 0.98).

Due to the short sampling period, *c*_DGT_ values are unsuited for predicting the P quantity that plants can take up from soil, whereas long-term sink extractions perform well for this purpose. 24-hour *c*_DGT_ values are, however, good predictors of P deficiency and relative yield (Degryse et al. 2009; Mason et al. 2010). A plot of a yield parameter, plant biomass production, against *c*_DGT_ (Fig. 3d), yields a saturation-type correlation similar to the findings of Mason et al. (2010). The six datapoints of the present study are, however, clearly insufficient for a detailed comparison with literature data. Nevertheless, the dependence of *c*_DGT_ on *k*_1_ shows that the improvement in yield prediction by *c*_DGT_ compared to standard P tests like Colwell P, Bray P, Olsen P etc. (Mason et al. 2010; Menzies et al. 2005; Six et al. 2013) is achieved because short-term DGT applications take P desorption kinetics into account. *P*_max_ plotted against plant biomass yields a similar relationship (Fig. 3c), indicating that *P*_max_ might generally also be used for yield prediction, although due to the high effort of its determination, this will have more relevance for research and calibration of soil P tests than for routine application.

## Conclusions

Our data indicates that in some soils no slow-release P fraction might exist, contrary to the existing understanding of soil P desorption. Our data is however not sufficient for conclusively elucidating this observation, therefore further work on a larger set of soils with different properties and documented, effective biocidal treatments should be carried out.

Moreover we show that the P quantity extracted by infinite P sinks in short-term and long-term applications are closely related to plant yield (biomass production) and plant P uptake. Infinite-sink extractions may therefore be a valuable research tool for reliably quantifying the plant available soil P fraction. It might also be an interesting tool for distinguishing P that is plant-available solely through abiotic desorption and dissolution from the effect of biotic P solubilisation (e.g. carboxylate exudation, microbial degradation). Lastly, long-term infinite sink-extracted P could serve as a reference method for the development of novel soil P tests.
